# Lipid Disorders in NAFLD and Chronic Kidney Disease

**DOI:** 10.3390/biomedicines9101405

**Published:** 2021-10-06

**Authors:** Meng Yang, Chang-An Geng, Xinguang Liu, Min Guan

**Affiliations:** 1 Guangdong Provincial Key Laboratory of Medical Molecular Diagnostics, Institute of Biochemistry and Molecular Biology, Institute of Aging Research, Guangdong Medical University, Dongguan 523808, China; yangmeng@gdmu.edu.cn; 2 Center for Human Tissues and Organs Degeneration, Institute of Biomedicine and Biotechnology, Shenzhen Institute of Advanced Technology, Chinese Academy of Sciences, Shenzhen 518055, China; 3 State Key Laboratory of Phytochemistry and Plant Resources in West China, Kunming Institute of Botany, Chinese Academy of Sciences, Kunming 650201, China; gengchangan@mail.kib.ac.cn

**Keywords:** lipid, NAFLD, chronic kidney disease

## Abstract

Nonalcoholic fatty liver disease (NAFLD) is the most common cause of chronic liver dysfunction and is characterized by exaggerated lipid accumulation, inflammation and even fibrosis. It has been shown that NAFLD increases the risk of other chronic diseases, particularly chronic kidney disease (CKD). Lipid in excess could lead to liver and kidney lesions and even end-stage disease through diverse pathways. Dysregulation of lipid uptake, oxidation or de novo lipogenesis contributes to the toxic effects of ectopic lipids which promotes the development and progression of NAFLD and CKD via triggering oxidative stress, apoptosis, pro-inflammatory and profibrotic responses. Importantly, dyslipidemia and release of pro-inflammatory cytokines caused by NAFLD (specifically, nonalcoholic steatohepatitis) are considered to play important roles in the pathological progression of CKD. Growing evidence of similarities between the pathogenic mechanisms of NAFLD and those of CKD has attracted attention and urged researchers to discover their common therapeutic targets. Here, we summarize the current understanding of molecular aberrations underlying the lipid metabolism of NAFLD and CKD and clinical evidence that suggests the relevance of these pathways in humans. This review also highlights the orchestrated inter-organ cross-talk in lipid disorders, as well as therapeutic options and opportunities to counteract NAFLD and CKD.

## 1. Introduction

Nonalcoholic fatty liver disease (NAFLD) has replaced viral liver diseases as the leading cause of chronic liver disease, with a worldwide prevalence of 25% [1]. NAFLD is characterized by excessive fat accumulation in hepatocytes and may progress to non-alcoholic steatohepatitis (NASH), ultimately leading to advanced fibrosis and cirrhosis [2]. Hepatic steatosis adversely affects multiple organs, placing abnormal lipid metabolism associated with NAFLD in close relation to many of the current life-style-related diseases [3]. It has been shown that NAFLD is part of a multisystem disease and is considered as a risk factor for extra-hepatic chronic complications, including type 2 diabetes mellitus (T2DM), cardiovascular disease (CVD) and chronic kidney disease (CKD) [4]. CKD is defined by abnormalities of kidney structure or function which are assessed using a matrix of variables including glomerular filtration rate, thresholds of albuminuria and duration of injury [5]. The prevalence of CKD is estimated to be 8–16% worldwide [6] and it increases to 23.4–35.8% in patients over 64 years old [7]. Patients with CKD are likely to die prematurely before progressing to end-stage renal disease (ESRD) [8]. The leading cause of death in these patients is CVD, which might be induced by dyslipidemia, hypertension, diabetes mellitus, or other factors [9].

Due to the rise in global epidemics of obesity and T2DM, the incidences of NAFLD and CKD have rapidly grown during recent decades [10]. Recently, increasing attention has been focused on NAFLD-related CKD. Emerging data have highlighted a strong correlation between NAFLD and CKD. NAFLD patients are more likely to have a higher urinary albumin excretion rate [11]. A meta-analysis reported that the risk of CKD in NAFLD patients is approximately two-fold higher than non-NAFLD patients [12,13]. Furthermore, NASH and advanced fibrosis are associated with a higher prevalence and incidence of CKD than simple steatosis [12]. Notably, growing evidence has shown that ectopic lipid deposition plays a critical role in accelerating the progression of NAFLD and CKD [14,15]. These clues suggest that NAFLD might be an important risk factor of CKD. As such, a better understanding of NAFLD and CKD pathogenesis regulated by lipid disorder is valuable in the search for novel therapeutic targets for NAFLD and CKD.

Previous reviews indicated that the liver and kidney share a number of pathways that are intrinsically linked to each other and provided an integrated summary of potential mechanisms of NAFLD involvement in CKD [13,16,17]. However, the effects of lipid metabolism in these two diseases are not described in detail. Here, we provide some putative molecular mechanisms of lipid accumulation in the liver and kidney and the pathogenesis of NAFLD and CKD deriving from toxic effects of excess lipids. We further emphasize the current understanding of inter-organ cross-talk between the liver and kidney in lipid metabolism. Finally, we summarize several promising therapies for prevention and treatment of NAFLD and CKD.

## 2. Molecular Mechanisms of Hepatic and Renal Lipid Accumulation

**Figure 1:**
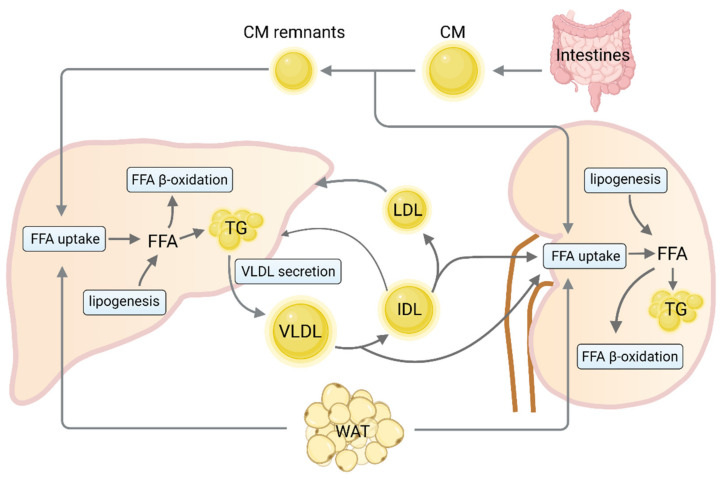
Cross-talk between liver and kidney in lipid metabolism. Dietary fat is incorporated into CM in the intestine and enters the circulation within two hours after food intake to deliver fatty acids to the kidney before being taken up by the liver as chylomicron remnants. In fasting state, FFA are derived from lipolysis in WAT and are actively taken up by various FA transporters. FFA derived from de novo lipogenesis or circulating are esterified to predominantly produce TG stored within lipid droplets. TG in the liver is packed into VLDL particles and exported into the blood stream for the delivery of fat to the kidney. Alternatively, fatty acids can be oxidized, primarily via β-oxidation, for energy production in the liver and kidney. This figure was created with BioRender.com (accessed on 2 October 2021). CM, chylomicrons; FFA, free fatty acid; WAT, white adipose tissue; TG, triglycerides; VLDL, very low-density lipoproteins; IDL, intermediate-density lipoprotein; LDL, low-density lipoprotein.

Numerous studies have demonstrated that dysregulation of lipid homeostasis is strongly associated with fatty liver [18,19]. In individuals with NAFLD, hepatic lipid accumulation is a consequence of lipid acquisition exceeding lipid disposal. This arises from the disruption of one or more of four major pathways: circulating lipid uptake, de novo lipogenesis, fatty acid oxidation and export of lipids in very low-density lipoproteins (VLDL). Once uptake/production of lipid breaks the equilibrium with oxidation/export, an unsteady state of liver lipid is progressed [20]. Abnormal renal lipid metabolism has also been described in an abundance of animal models with renal injury [21]. Similar to liver, molecular mechanisms responsible for lipid accumulation in the kidney are also associated with dysregulation of multiple lipid metabolism pathways (Figure 1).

Circulating free fatty acid (FFA) can be generated from the absorption of dietary fat or lipolysis of triglycerides (TG) stored in white adipose tissue. Uptake of circulating FFA is largely dependent on both the concentration of plasma fatty acids and the capacities of membrane-bound fatty acid transport proteins (FATPs), as well as cluster of differentiation 36 (CD36) [22]. It is well known that circulating FFA pool in an obese state is held accountable for the majority of liver lipids in NAFLD [23]. Meanwhile, localization of CD36 on the plasma membrane of hepatocytes and CD36 palmitoylation are markedly increased in the liver of mice with NASH, enhancing the uptake of FFA [24]. However, knockdown of FATP2 or FATP5 in mice reduces the hepatocyte fatty acid uptake and ameliorates hepatic steatosis induced by a high-fat diet (HFD) [25,26]. Given the high volume of blood that passes through, the kidney is also easily affected by the amount of circulating FFA from lipolysis in adipocytes [27]. In vivo data suggest that FATP2 regulates proximal tubule apical non-esterified fatty acids (NEFA) uptake and could be the crucial inciting factor for kidney fibrosis development [28,29]. Besides, CD36 knockout mice fed an HFD displayed lower renal lipid accumulation and had less glomerular and tubulointerstitial macrophage accumulation, foam cell formation, oxidant stress and interstitial fibrosis [30]. These data indicate that excess fatty-acid transport into the liver and kidney is required for NAFLD and CKD.

Together with elevated lipid influx, an increase in de novo lipid synthesis aggravates hepatic lipid accumulation. A stable isotope study demonstrated that 26% of hepatic lipid content in patients with NAFLD is derived from de novo lipogenesis [31]. Previous studies have demonstrated the important roles of steroyl-CoA response element binding protein-1c (SREBP-1c) and carbohydrate response element-binding protein (ChREBP) in the development of hepatic steatosis due to the increase in transcription of enzymes involved in de novo lipogenesis, including acetyl-CoA carboxylase 1 (ACC1), fatty acid synthase (FASN) and stearoyl-CoA desaturase 1 (SCD1) [32,33]. In addition, fructose, a commonly consumed sugar in Western diet, also significantly upregulates the expression of SREBP-1c and other lipogenic enzymes contributing to lipid disorders [34]. Growing evidence supports that lipogenesis-related SREBP-1c and ChREBP transcription factors also contribute to an increase in TG content in cultured tubular cells [35,36]. Overexpression of ChREBP significantly drives reactive oxygen species (ROS) production which may cause renal tubular damage [37]. Meanwhile, renal TG accumulation can be prevented in the kidneys of SREBP1c-defienced mice [38]. As noted above, SREBP-1c and ChREBP are necessary for de novo lipogenesis-induced hepatic or renal lipid accumulation.

Dysregulated expression of enzymes involved in FFA β-oxidation also contributes to hepatic and renal lipid accumulation. A liver-specific defect in adipose triglyceride lipase (ATGL) or carnitine palmitoyltransferase 2 (CPT2) results in steatosis and the loss of both components leads to significant steatohepatitis upon high-fat feeding [39]. The activation of peroxisome proliferator-activated receptor α (PPARα) induces the transcription of genes related to mitochondrial fatty acid oxidation, such as carnitine palmitoyltransferase 1A (*CPT1A*) and acyl-CoA oxidase 1 (*ACOX1*), thereby reducing lipid accumulation [40]. PPARα expression is downregulated in patients with NASH and negatively correlates with NASH severity [41]. Furthermore, PPARδ also attenuates hepatic steatosis by stimulating autophagy-mediated fatty acid oxidation [42]. In vitro experiments indicated that the inhibition of fatty acid oxidation by CPT1 inhibitor etomoxir in proximal tubular cells led to ATP depletion, cell death, dedifferentiation and intracellular lipid deposition, all of which are phenotypes observed in fibrosis [43]. Additionally, aging has been found to aggravate renal lipid accumulation and fibrosis by impairment of PPARα and the fatty acid oxidation pathway [44]. A longitudinal study that included 92 American Indians with T2DM with preserved glomerular filtration rate suggested impaired fatty acid β-oxidation may contribute to the progression of diabetic kidney disease [45]. More importantly, genome-wide transcript data from a large cohort of kidney samples from individuals with CKD confirmed the strong enrichment for fatty acid β-oxidation among the differently expressed pathways [43]. Hence, PPARs are considered as potential therapeutic targets that alleviate intracellular lipids accumulation via enhancing lipid oxidation.

Apart from mitochondrial β-oxidation, the other major fate of fatty acids in hepatocytes is re-esterification to form TG, which can be exported into the blood as a VLDL particle. The secretion of TG-enriched VLDL (VLDL-TG) from the liver plays an essential role in regulating intrahepatic and circulating lipid homeostasis [46]. Impaired VLDL assembly and secretion is a key factor for developing hepatic steatosis and NASH pathogenesis [47,48]. The suppression of hepatic expression of apolipoprotein B (ApoB) and microsomal triglyceride transfer protein (MTP) required for VLDL biogenesis leads to limiting VLDL-TG export and increased hepatic TG accumulation [49]. Cell death-inducing DFF45 like effector B (CIDEB) [50] and phospholipase A2 group XIIB (PLA2G12B) [51,52] also play critical roles in modulating hepatic VLDL-TG secretion and lipid homeostasis. Dysregulation of VLDL-TG secretion has been demonstrated to cause atherogenic dyslipidemia and renal lipid accumulation [53,54,55]. However, whether hepatic VLDL-TG secretion is associated with the pathogenic progression of CKD remains to be further investigated.

Taken together, lipidosis in liver and kidney is a consequence of an imbalance between the influx of fatty acids, lipid synthesis, oxidation and export, which has been implicated in the pathogenesis of NAFLD and CKD.

## 3. Lipid Disorders Contribute to Pathogenic “Cross-Talk” between NAFLD and CKD

Experimental and epidemiological data reveal some pathophysiological links between them and support the assertion that NAFLD may be a pathogenic factor of CKD [12,13], wherein CKD accelerates the progression of NAFLD [56]. Among these, several mechanisms of action by which lipids can cause liver and renal damage have been proposed. It has been generally accepted that the generation of lipotoxic metabolites of fatty acids typically occurred in parallel with lipid accumulation, which plays a critical role in the pathogenesis of NAFLD and CKD. Lipotoxicity predisposes liver to excessive ROS production [57,58] and oxidative stress which may cause membrane lipid peroxidation, cell necrosis and cell death by apoptosis [59,60]. It has been suggested that alterations in the lipid metabolism significantly alter mitochondrial functions in the context of diabetic kidney disease [61], as well as in patients and animal models of NAFLD [62,63]. For example, mitochondrial dysfunction leads to a systemic inflammatory response due to liver injury [63]. The pathogenesis of NAFLD seems to be a vicious cycle of steatosis, lipotoxicity and inflammation resulting in a gradual decline of the biological functions of the liver [64]. Specifically, an overload of FFA into mitochondria may contribute to an increase in the permeability of the inner mitochondrial membrane, which leads to the loss of membrane potential and ATP synthesis capacity, resulting in mitochondrial dysfunction [65]. The initial mitochondrial function impairment can be further amplified by the production of mtDNA mutation by ROS [65]. ROS are important mediators of lipotoxicity-induced injury of visceral glomerular epithelial cells that are essential for maintaining the glomerular tuft and filtration barrier [66]. Moreover, ROS may promote the expression of profibrotic molecules, such as transforming growth factor-beta 1 (TGF-β1), therefore playing a major role in the development of renal fibrosis, a progressive and usually irreversible process, causing CKD [67].

Recent evidence shows that endoplasmic reticulum (ER) stress induced by lipid overload has been widely involved to drive NAFLD progression, as well as kidney injury [68,69]. Activation of the unfolded protein response (UPR) was observed in the livers of experimental obese models, as well as obese humans with NASH [70,71]. ER stress also induces proinflammatory signaling in hepatocytes, thus contributing to inflammation-mediated liver injury in chronic liver diseases [72] and in renal culture cells [73]. Treatment with saturated fatty acid and palmitic acid activated UPR by upregulation of the ER chaperone binding immunoglobulin protein (BIP), transcription factor 4 (ATF4) and proapoptotic transcription factor C/EBP homologous protein (CHOP), protein in cultured human proximal tubule epithelial cells [74]. Prolonged ER stress resulted in enhanced apoptosis of lipid-enriched proximal tubule cells with colocalization of BIP and SREBP-2 [75]. In addition, ER stress has been causally linked to the development of renal insulin resistance through c-jun N-terminal kinase (JNK) activation and inflammation [76]. A study performed in cultured human glomerular mesangial cells has shown that the inhibition of ER stress by 4-phenylbutyrate markedly suppressed inflammatory cytokine secretion [77]. Based on the mechanistic view described above, mitochondrial dysfunction, ER stress and ROS resulting from intracellular lipid overload play an important role in development of NAFLD, as well as CKD.

On the other hand, lipid metabolism dysfunction is associated with insulin resistance that is considered as a key pathogenic factor in NAFLD and CKD. There is evidence that increased levels of serum FFA, elevated pro-inflammatory cytokines, lower adiponectin levels or an increase in de novo lipogenesis in patients with NAFLD play a central role in mediating insulin resistance [78]. Furthermore, an excess of intrahepatic molecules, such as diacylglycerols (DAGs) and ceramides, are shown to promote hepatic insulin resistance, activate hepatic stellate cells and increase the production of the collagen matrix leading to the progression of liver disease [17]. Meanwhile, HFD or palmitic acid overload leads to the upregulation of inflammation, fibrosis, or cell death in kidneys [79,80]. Specifically, treatment with palmitic acid promotes insulin resistance and changes in the cytoskeleton, leading to apoptosis in cultured podocytes [81]. Furthermore, clinical data support that preserved insulin signaling in the glomerular podocyte is an important contributor to normal kidney function [82]. However, disturbance of insulin signaling was observed in individuals with mild, advanced, or end-stage CKD and may directly contribute to the development of diabetic kidney disease [82,83].

Hepatic lipid accumulation in NAFLD induces dyslipidemia by increasing the secretion rate of VLDL [49] and then impacts extrahepatic tissues. VLDL exchanges TG with the cholesterol contained in circulating low-density lipoprotein (LDL) and high-density lipoprotein (HDL), resulting in the formation of small LDL (sLDL) and reduced level of small HDL cholesterol (HDL-C) particles [84]. Coincidentally, dyslipidemia, in the majority of CKD patients, is usually characterized by high LDL cholesterol (LDL-C), low HDL-C and high TG levels [85,86]. LDL levels strongly correlated with lipid contents and fibrosis in grafted kidneys of patients with CKD [87]. The accumulation of oxidized sLDL particles causes renal damage by triggering glomerular injury, mesangial cell proliferation and foam cell formation [56,88]. Furthermore, clinical and experiment data have shown that low HDL-C levels were a risk factor for the development of renal dysfunction [89,90]. HDL possesses key antioxidant and anti-inflammatory properties which play a crucial role in the protection against foam cell formation by preventing oxidation of LDL and activation of leukocyte and endothelial cells [91,92]. Significantly lower HDL levels in NAFLD, especially NASH patients [93], may act as a driver of CKD [91]. Additionally, uric acid, ROS and toxic metabolites derived from NAFLD also play crucial roles in the development of CKD [17].

Moreover, liver-specific effects on extrahepatic complications could be mediated by secretion of multiple inflammatory cytokines, such as C-reactive protein (CRP), tumor necrosis factor alpha (TNF-α) and interleukin 6 (IL-6), or hepatokines, such as fetuin-A, fibroblast growth factor 21 (FGF21) and insulin-like growth factor 1 (IGF-1) [13]. Especially, inflamed liver modulates whole-body metabolism and inflammation via CRP, TNF-α and IL-6 [56]. Fetuin-A is secreted exclusively by hepatocytes in response to ER stress [94] and suppresses adiponectin release by adipocytes [95]. Of note, adiponectin was shown to attenuate renal injury and fibrosis in a mouse model of CKD [96]. FGF21 has been demonstrated to attenuate kidney injury in CKD [56,97]. However, there is an impaired action of FGF21 in NAFLD, although its systemic levels are elevated [98]. Additionally, IGF-1 levels are inversely related to the severity of liver injury and crucial for podocyte cell function, thereby maintaining glomerular filtration rate in CKD patients [99]. These effects suggest that NAFLD affects renal injury mainly through lipoprotein dysmetabolism and altered secretion of hepatokines.

Accumulating clinical evidence in recent years indicated an increased risk of NAFLD in CKD patients [100,101]. Kidney dysfunction affects NAFLD/NASH pathogenesis mainly through ROS, systemic inflammation, modulating gut microbiota and uremic toxins, as well as renin-angiotensin system (RAS). Above all, gut microbiota modulates the severity of chronic liver damage [102]. The alterations in the composition and function of gut microbiota during the progression of CKD induce leakage of endotoxins, leading to the activation of receptor-mediated immune cells, release of pro-inflammatory cytokines in the circulation and subsequent inflammation within the liver [103,104]. Gut microbiota and intestinal dysbiosis occurring in CKD result in the formation of short-chain fatty acids (SFCAs), which contribute to the development of liver adiposity and hepatic insulin resistance [105,106]. Accumulation of uremic toxins in the circulation is a common accompaniment to CKD [107]. Notably, the incubation of primary human hepatocytes with uremic toxins significantly downregulated bile acid uptake transporters and interfered with mitochondria function [107]. Furthermore, both the kidney and liver express RAS constituents, the activation of which plays a key role in the pathogenesis of NAFLD and CKD by elevating insulin resistance, oxidative stress and pro-inflammatory cytokine production [16].

**Figure 2:**
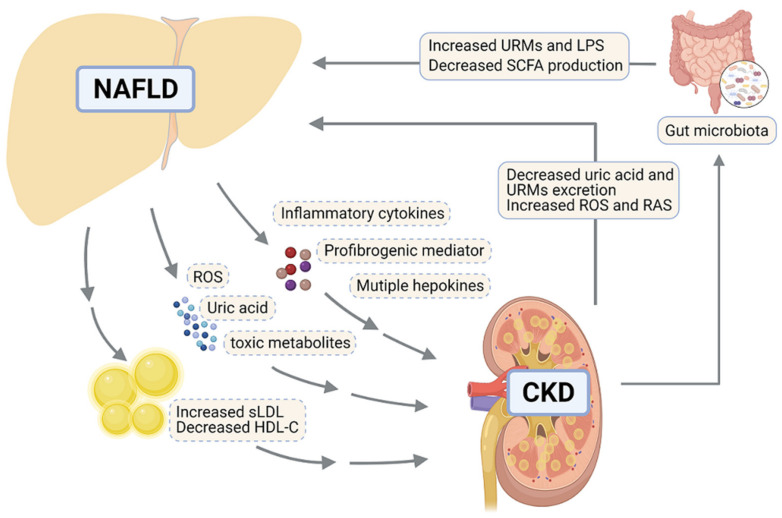
Molecular pathways mediating the interactions between liver and kidney in promoting NAFLD and CKD. In NAFLD, the steatotic and inflamed liver releases inflammatory cytokines including TNF-α and IL-6, profibrogenic mediator and multiple hepatokines (e.g., FGF21), contributing to impaired kidney functions. Additionally, the liver promotes CKD through overproducing uric acid, ROS, certain toxic metabolites and VLDL particles, which promotes atherogenic dyslipidemia through increased sLDL and decreased HDL-C. CKD contributes to NAFLD via reduced excretion of uric acid and URMs, as well as increased ROS and RAS. Furthermore, in CKD, the kidney connects to the pathogenic processes of NAFLD by modulating gut microbiota composition, which enhances the level of URMs, LPS and SCFA. This figure was created with BioRender.com (accessed on 2 October 2021). NAFLD, nonalcoholic fatty liver disease; CKD, chronic kidney disease; sLDL, small low-density lipoprotein; HDL-C, high-density lipoprotein-cholesterol; ROS, reactive oxygen species; RAS, renin-angiotensin system; URMs, uremic retention molecules; LPS, lipopolysaccharide; SCFA, short-chain-fatty acid; TNF-α, tumor necrosis factor alpha; IL-6, interleukin 6; FGF21, fibroblast growth factor 21.

The findings reported above not only provide key insights regarding the underlying mechanism linking lipid abnormalities to NAFLD and CKD progression, but also suggest that lipids mediate the pathogenic “cross-talk” between these two diseases. Figure 2 summarizes the risk factors potentially linking NAFLD and CKD. The complex link between NAFLD and CKD suggests that multi-targeted therapies could help in the complicated context.

## 4. Common Therapeutic Strategies for NAFLD and CKD

There are currently no approved treatments for NAFLD/NASH, whereas, novel drugs have been approved for CKD [108]. Increasing our knowledge on lipid metabolism in the liver and kidney, as well as their inter-organ cross-talk, could reveal mechanistic insights necessary for the development of new therapeutic strategies for NAFLD/NASH and CKD. Here, we highlight some potential therapeutic targets for the prevention and treatment of NAFLD and CKD (Table 1).

### 4.1. Lipid Mediators as Optional Therapeutic Targets

Sodium-glucose co-transporter-2 (SGLT2) inhibitors appear to represent a promising option for the management of NAFLD and CKD. SGLT2 is expressed almost exclusively in the epithelial cells of the proximal convoluted tubule and mediates approximately 90% of the active renal glucose reabsorption [109]. SGLT2 inhibitors not only play an important role in improving systemic glucose homeostasis but also have protective effects on the kidney in individuals with T2DM [110]. SGLT2 inhibitors, such as dapagliflozin, empagliflozin and canagliflozin, induce a significant reduction in albuminuria in patients with T2DM and CKD [111]. In a clinical trial setting, canagliflozin treatment in individuals with diabetic kidney disease decreased the levels of inflammation and fibrosis biomarkers, including IL-6, matrix metallopeptidase 7 (MMP7) and fibronectin 1(FN1) [112]. Importantly, dapagliflozin is approved in the European Union for treating CKD in adults with and without diabetes [108]. Furthermore, weight loss and increased fatty acid oxidation during the administration of SGLT2 inhibitors could contribute to the reduction of hepatic fat accumulation in patients with T2DM and NAFLD [113]. Empagliflozin could significantly ameliorate liver injury in an animal model of T2DM with NAFLD, through enhancing hepatic macrophage autophagy via the AMP-activated protein kinase/mechanistic target of rapamycin (AMPK/mTOR) signaling pathway and further inhibiting hepatic inflammatory responses [114]. SGLT2 inhibitor NGI001 blocked de novo lipogenesis by substantially suppressing the expression of FASN and SREBP-1c, promoted fatty acid β-oxidation through increasing the expression of ATGL, CPT1 and PPARα and thus alleviated fat droplet accumulation in a cell model of human fatty liver [115].

An extensive deregulation of nuclear transcription factors is responsible for lipid abnormalities. It has been revealed that PPARs and farnesoid X nuclear receptor (FXR) are downregulated in NAFLD and CKD [116,117,118,119]. PPARs, as members of the steroid hormone receptor superfamily, play key roles in the transcriptional regulation of fatty acid metabolism, inflammation and fibrogenesis [120,121]. On this basis, PPAR ligands are considered as promising therapeutic agents for NAFLD and CKD. For instance, lanifibranor is a pan PPAR agonist that activates all three subtypes (α, β/δ and γ), giving it the effectiveness to trigger the resolution of steatohepatitis and regression of fibrosis [116]. A PPARα agonist, WY14643, seems to prevent tubule cell death and intracellular lipid accumulation [122]. PPARγ agonist rosiglitazone causes the reduction in hepatic steatosis through improving FFA metabolism [123]. As an endogenous ligand of PPARγ, 15d-PGJ2 prevents renal fibrosis in rats undergone unilateral ureteral occlusion surgery leading to renal dysfunction [124]. The activation of FXR locally in the liver has been shown to be protective against the development of hepatic steatosis and NASH via affecting multiple cell types in the liver [125]. FXR agonist obeticholic acid effectively reduced serum TG levels, alanine aminotransferase (ALT) and markers of liver fibrosis in patients with NAFLD and T2DM in a phase II study [126]. Patients with NASH who received obeticholic acid showed improved liver histology (2-point or greater improvement in NAFLD activity score without worsening of fibrosis) [118]. On the other hand, treatment of FXR agonist GW4064 in a type 2 diabetic kidney animal model ameliorated albuminuria, pro-fibrotic and pro-inflammatory changes, improved renal lipid metabolism and inhibited renal autophagy, apoptosis and ROS production, suggesting that FXR may also be a therapeutic target for CKD [127,128].

Selective thyroid hormone receptor β (TRβ) agonists also provide new perspectives for the treatment of NAFLD and CKD. The thyroid hormone mediates hepatic lipogenesis, fatty acid β-oxidation, cholesterol synthesis and reverse cholesterol transport [129]. Treatment with TRβ agonist resmetirom decreased hepatic steatosis and circulating lipids and repressed fibrogenic genes expression in mice fed a diet high in fat, fructose and cholesterol for 34 weeks [130,131]. A clinicopathological study shows that nephrotic syndrome patients with a thyroid dysfunction have higher urine protein and lipid levels than those with normal thyroid function [132]. TRβ has also been shown to be expressed in tubule cells of CKD patients and take part in the regulation of cell-cycle progression in renal tubule epithelial cells [133]. These findings support the potential effect of selective TRβ agonists to improve liver and kidney injury.

Recently, biological agents targeting the proprotein convertase subtilisin-kexin type 9 (PCSK9) have been shown to reduce LDL-C by 50–60% [134] and alleviate NAFLD [135]. A study indicates that high intrahepatic or circulating PCSK9 levels increase liver lipid storage and secretion, thus contributing to the pathogenesis of NAFLD [136]. PCSK9 inhibitor therapy significantly ameliorates steatosis biomarkers such as the hepatic steatosis index in familial hypercholesterolemia patients with low TG/HDL [137]. In CKD, increased plasma lipid levels are associated with elevated levels of PCSK9, suggesting a role for PCSK9 in CKD-associated dyslipidemia [138]. Given the limited efficacy of statins in CKD, further research could focus on therapeutically targeting PCSK9 to treat CKD.

Based on their important roles in lipid metabolism, CD36, FATP2 and FGF21 are also considered as potential therapeutic targets for NAFLD and CKD [30,139,140]. Additionally, miRNAs might be molecular targets for metabolic regulation of NAFLD or CKD [141,142,143,144], the delivery and application of which require further investigation. Furthermore, studies in the past two years provided novel insights into several promising candidates such as selective peroxisome proliferator-activated receptor alpha modulator (SPPARMα), 15-lipoxygenase (Alox15) and cAMP-responsive element-binding protein H (CREBH); it is still necessary to discover more potential molecular targets for the prevention and treatment of NAFLD and CKD.

**Table 1:** Roles of potential therapeutic targets/strategies in the pathogenesis of NAFLD and CKD.

Targets/Strategies	Mechanism of Action	Effect on NAFLD	Effect on CKD	Drug Candidates	Ref.
SGLT2	Mediates renal glucose reabsorption	Inhibiting SGLT2 ameliorates NAFLD	Inhibiting SGLT2 ameliorates CKD	Dapagliflozin is approved for treating CKD; Empagliflozin: Phase 4 recruiting (NASH), NCT04639414	[108,109,110,111,112,113,114,115]
PPARs	Induce fatty acid β-oxidation via inducing the transcription of CPT1A and ACOX1 Reduce TG synthesis and insulin resistance Inhibit inflammatory cell activation and fibrotic processes	Improve hepatic steatosis and NASH	Prevent renal fibrosis and dysfunction	Lanifibranor: Phase 2 completed (NASH), NCT03008070; Pioglitazone: Phase 2 recruiting (NASH), NCT04501406	[40,42,117,120,121,122,123,124,145]
FXR	Decreases lipogenesis by down-regulating SREBP1c Regulates bile acid homeostasis Enhances insulin sensitivity Decreases autophagy and apoptosis Reduces inflammation and fibrosis	Protects against the development of hepatic steatosis and NASH	Prevents the progression of acute kidney injury to CKD Improves diabetic nephropathy	Obeticholic acid: Phase 3 (NASH), NCT02548351; Tropifexor: Phase 2 recruiting (NASH), NCT04065841; EYP001a: Phase 1 completed (NASH), NCT03976687	[52,118,125,126,127,128,145]
TRβ	Stimulates expression of CPT1A and fatty acid β-oxidation Decreases lipogenesis by attenuating expression of SREBP1c Reduces serum levels of LDL cholesterol	Prevents hepatic steatosis	May prevent cell death in early stage of kidney injury	Resmetirom: Phase 3 recruiting (NAFLD), NCT04951219	[129,130,131,132,133,145]
PCSK9	Reduces cell surface LDL receptor concentration and increases circulating LDL-C significantly Increases VLDL secretion by inducing ApoB and MTTP expression Increases insulin resistance Increases de novo lipogenesis by upregulating PPARγ, SREBP1 and FASN	Induces hepatic steatosis	Associates with CKD-related dyslipidemia	Evolocumab and Alirocumab are approved for lipid lowering therapy	[134,135,136,137,138,145,146]
Modulation of gut microbiome	Decreases intracelluar lipid accumulation, Reduces pro-inflammatory cytokin Affects bile acid production Suppresses the production of uremic toxins Improves urea utilization	Prevents and improves NAFLD	Reduces kidney injury	-	[147,148,149,150,151,152,153,154,155,156,157,158]
MSC transplantation	Inhibits immune responses Decreases cell apoptosis and fibrosis Promotes tissue regeneration and regeneration	Improves liver function and NASH	Reduces kidney injury and promotes renal repair	-	[159,160,161,162,163,164,165,166]

### 4.2. Novel Therapeutic Strategies

Lifestyle modifications might help prevent or slow the progression of NAFLD and CKD. Environmental factors such as high fat intake, excessive fructose consumption and vitamin D deficiency can promote the pathogenesis of both NAFLD and CKD [81,167]. It has been proven that all these factors can affect the gut microbiome and impair intestinal immunity [168,169]. New therapeutic approaches for modulating gut microbiota have been proposed to prevent and improve NAFLD. Comparisons of germ-free and conventional mice exhibit that gut microbiota prevents fibrosis upon chronic liver injury [149,156]. HFD mice that underwent fecal microbiota transplantation for eight weeks presented a noticeable improvement of steatohepatitis via a significant reduction in intrahepatic lipid accumulation and pro-inflammatory cytokines (e.g., Interferon -γ and IL-17) [147]. In another study using a mouse model, probiotics treatment protected against the fructose-induced liver steatosis by attenuating Toll-like receptor 4 (TLR4) signaling in the liver [150,151]. Noteworthy, analyses on clinical data of NAFLD patients show that probiotic mixtures can reduce the levels of ALT and aspartate aminotransferase (AST), reduce liver fat and inflammatory cytokines [153,154]. Perturbation of the composition of gut microbiota has also been observed in patients suffering from CKD [157,158]. Although there are few data about fecal microbiota transplantation for the treatment of CKD, interventions designed to restore the imbalance of the gut-kidney symbiosis are possible treatment options. For instance, supplementation with prebiotic lactulose modifies gut microbiota and suppresses the production of uremic toxins, leading to ameliorated renal function in adenine-induced CKD rats [155]. Probiotics also reduce kidney injury by restoring gut microbiota and improving urea utilization [148,152]. Therefore, the modulation of the gut microbiome composition may be an effective and safe therapeutic strategy for NAFLD and CKD.

In recent years, mesenchymal stem cells (MSCs)-based therapy has gradually become a hot topic for degenerative and inflammatory disorders, including kidney and liver diseases [162]. The ability of infused MSCs to resolve inflammation and promote renal repair has been demonstrated in various models of kidney diseases. Allogeneic bone marrow-derived MSCs (BM-MSCs) transplantation repressed immune responses and induced the remodeling of the extracellular matrix in rats with nephrectomy [163]. Additionally, exosomes derived from BM-MSCs were shown to improve diabetic nephropathy in mice by mediating the attenuation of renal inflammation, cell apoptosis and kidney fibrosis [166]. Adipose tissue-derived MSCs are potent in suppressing inflammation and cellular stress, promoting renal cell survival and ameliorating interstitial fibrosis in pig with renal artery stenosis [164,165]. On the other hand, MSCs therapy has been reported to effectively promote liver regeneration and repair liver injury in NAFLD. MSCs engrafted into the liver restored albumin expression in hepatic parenchymal cells, ameliorated fibrosis and impeded the number of intrahepatic-infiltrating immune cells in a NASH model [159]. MSCs transplantation reduced HFD-induced hepatic steatosis, lobular inflammation and liver fibrosis in mice with NAFLD [161]. BM-MSCs transplantation also alleviated CCl4-induced rat liver fibrosis by suppressing the levels of IL-17A accompanied by the downregulation of the IL6/signal transducer and activator of transcription 3 (STAT3) signaling pathway [160].

## 5. Conclusions

NAFLD and CKD are chronic, frequently progressive conditions that develop in response to sustaining fat accumulation, which is a result of lipid acquisition surpassing lipid disposal. In other words, increased circulating lipid uptake and lipogenesis mediate excessive lipid acquisition in the liver or kidney, while a compensatory enhancement of fatty acid oxidation or VLDL secretion is insufficient in normalizing lipid levels. Enhanced generation of ROS and oxidative stress, as a consequence of lipid overload, represent the primary cause of liver and renal injury. ER stress, mitochondrial dysfunction and insulin resistance further trigger cell apoptosis, inflammation and fibrosis in the liver and kidney. As an important risk factor for CKD, NAFLD can cause renal damage through the induction of atherogenic dyslipidemia and secretion of multiple hepatokines. In return, CKD may affect NAFLD/NASH pathogenesis through gut microbiota and RAS. Accumulating evidence indicates several potential therapeutic targets, including nuclear transcription factors. Moreover, novel therapeutic strategies involving gut microbiota and MSCs may also be promising approaches. In summary, a better understanding of lipid disorder regulated by inter-organ cross-talk between liver and kidney in different disease stages is valuable in the search for novel therapeutic targets for NAFLD and CKD. Nonetheless, the impact of lipid disorder on CKD and NAFLD needs more insights from large-scale prospective studies, paving ways for developing new therapeutic targets.
